# From Guidelines to Practice: Implementing Human Rights and Recovery Approaches in Specialist Trauma Services

**DOI:** 10.1192/j.eurpsy.2026.11877

**Published:** 2026-07-31

**Authors:** P. McGovern

**Affiliations:** Traumepoliklinikken, Modum Bad, Oslo, Norway

## Abstract

**Introduction:**

Human rights and recovery-based approaches are gaining global recognition as essential components of ethical mental health care. The World Health Organization’s QualityRights materials and policy guidance call for services to affirm autonomy, dignity, and the right to health — especially for individuals living with psychosocial disabilities. Yet, these principles are rarely applied in everyday clinical settings, particularly for patients with complex trauma histories, high symptom burden, and long-standing relational difficulties.

**Objectives:**

This presentation explores the implementation of a trauma-informed group model developed at Modum Bad’s Traumepoliklinikk: *Trauma-oriented Recovery Planning* (TORP). It aims to show how recovery principles & human rights — including the right to decide, legal capacity, & supported decision-making — can be operationalised in a specialised treatment setting. The objectives are to:

- Present a replicable model that integrates WHO guidance with clinical care
- Explore how TORP strengthens autonomy, agency, and therapeutic alliance
- Share qualitative feedback on its impact from both patient and clinician perspectives

**Methods:**

TORP is a psychoeducational and exploratory group offered to individuals with cPTSD and complex dissociative disorders. It draws directly from WHO QualityRights modules and integrates supported decision-making practices, including:

- Mapping personal recovery values and goals
- Exploring legal capacity and the right to decide
- Developing tools for advanced care planning and preference articulation
- Building skills for self-advocacy within the health system

The model works with trauma dynamics while supporting people to live a good life despite what they have experienced. Qualitative data is drawn from participant feedback across several groups, focusing on shifts in self-understanding, agency, and hope for the future.

**Results:**

Participants report increased clarity around their rights and a new sense of ownership over their recovery. Many describe TORP as the first time they felt seen as a whole person, not just a diagnosis. Learning about legal capacity and being invited to make meaningful treatment choices shifted their relationship to care. Clinicians note increased trust, improved collaboration, and stronger therapeutic foundations for ongoing work. Advanced planning tools have proved useful for avoiding crisis-driven interventions and promoting shared understanding.

**Conclusions:**

TORP shows that human rights-based, recovery-oriented care is not only possible but clinically valuable — even in the context of severe and long-standing trauma. By integrating supported decision-making, affirming legal capacity, and offering space for reflection and planning, the model strengthens both patient agency and therapeutic outcomes. TORP offers a structured way to support people in reclaiming agency, building capacity, and shaping care in line with their values.

**Disclosure of Interest:**

None Declared

